# Building two *indica* rice reference genomes with PacBio long-read and Illumina paired-end sequencing data

**DOI:** 10.1038/sdata.2016.76

**Published:** 2016-09-13

**Authors:** Jianwei Zhang, Ling-Ling Chen, Shuai Sun, Dave Kudrna, Dario Copetti, Weiming Li, Ting Mu, Wen-Biao Jiao, Feng Xing, Seunghee Lee, Jayson Talag, Jia-Ming Song, Bogu Du, Weibo Xie, Meizhong Luo, Carlos Ernesto Maldonado, Jose Luis Goicoechea, Lizhong Xiong, Changyin Wu, Yongzhong Xing, Dao-xiu Zhou, Sibin Yu, Yu Zhao, Gongwei Wang, Yeisoo Yu, Yijie Luo, Beatriz Elena Padilla Hurtado, Ann Danowitz, Rod A. Wing, Qifa Zhang

**Affiliations:** 1 National Key Laboratory of Crop Genetic Improvement, Huazhong Agricultural University, Wuhan 430070, China; 2 Arizona Genomics Institute and BIO5 Institute, School of Plant Sciences, University of Arizona, Tucson, Arizona 85721, USA; 3 International Rice Research Institute, Genetic Resource Center, Los Baños, 4031 Laguna, Philippines

**Keywords:** Genomics, Plant genetics, DNA sequencing

## Abstract

Over the past 30 years, we have performed many fundamental studies on two *Oryza sativa* subsp. *indica* varieties, Zhenshan 97 (ZS97) and Minghui 63 (MH63). To improve the resolution of many of these investigations, we generated two reference-quality reference genome assemblies using the most advanced sequencing technologies. Using PacBio SMRT technology, we produced over 108 (ZS97) and 174 (MH63) Gb of raw sequence data from 166 (ZS97) and 209 (MH63) pools of BAC clones, and generated ~97 (ZS97) and ~74 (MH63) Gb of paired-end whole-genome shotgun (WGS) sequence data with Illumina sequencing technology. With these data, we successfully assembled two platinum standard reference genomes that have been publicly released. Here we provide the full sets of raw data used to generate these two reference genome assemblies. These data sets can be used to test new programs for better genome assembly and annotation, aid in the discovery of new insights into genome structure, function, and evolution, and help to provide essential support to biological research in general.

## Background & Summary

Rice is the leading staple crop for mankind and has been recognized as an important model organism for biological research, especially for monocot plants. Asian cultivated rice (*Oryza sativa*) is composed of two subspecies: *O. sativa* subsp. *japonica* and subsp. *indica*; *indica* rice accounts for over 70% of rice production worldwide^1^ and is genetically much more diverse^2^. The *indica* varieties Zhenshan 97 (ZS97) and Minghui 63 (MH63) represent two major varietal groups of *indica* rice^3^, contain a number of important agronomic traits and are the parents of Shanyou 63 (SY63), the most widely cultivated hybrid rice in China. The ZS97, MH63, SY63 hybrid system has been used as a model^4–9^ over the past 30 years, and concomitantly our lab has made a series of attempts to gain a fundamental understanding of the genetic basis of heterosis—a biological mystery that has puzzled the scientific community for more than 100 years. Hence, we initiated a joint collaborative project to generate two reference-quality genome assemblies for ZS97 and MH63 to be used as a fundamental tool to help us understand the underlying molecular genetic basis of heterosis^10^. In this descriptor, we report the resources and data sets that were generated and used to assemble the ZS97 and MH63 reference genomes: (1) two BAC libraries, (2) two improved physical maps and minimum tiling paths (MTP), (3) raw PacBio sequencing data of BAC pools and full clone sequence assemblies, (4) Illumina WGS sequence and assembly data, and (5) the first release of reference genome assemblies for ZS97 and MH63. With the resources and data generated in this study, we were not only able to *de novo* assemble two reference-quality genome sequences, but we were able to provide the scientific community with data to advance biological research at the genomic level, especially for further understanding of the genetic basis of heterosis.

## Methods

### BAC library construction and end-sequencing

The two BAC libraries used in this study were previously reported^11^. Briefly, partially digested (i.e., *Hin*dIII) and size-selected genomic DNA from each variety was cloned into the *Hin*dIII site of pAGIBAC1, and transformed into *Escherichia coli* DH10B T1-resistant competent cells^11^. Both libraries, named OSIZBa (ZS97) and OSIABa (MH63), contained 36,864 BAC clones, had average insert sizes were ~117 kb (ZS97) and ~125 kb (MH63), and covered ~10.0× (ZS97) and ~10.7× (MH63) of each genome^11^. Additionally, 33,969 (ZS97) and 35,549 (MH63) bi-directional BAC end sequences (BESs) were generated for the first half of each library^11^.

### Physical maps

Two low coverage physical maps (PMs), using the SNaPshot fingerprinting method, were described previously^11^. We rebuilt the two PMs using KeyGene’s Whole Genome Profiling (WGP) method^12^. WGP FingerPrint Contig (FPC) PMs were built in four steps: (1) BAC DNA preparation, (2) WGP preparation of BAC plasmids with indexing and sequencing adaptors, (3) Illumina sequencing, and (4) bioinformatic processing. In step 4, using WGP deconvolution scripts, 99,996 (ZS97) and 103,609 (MH63) unique WGP tags were deconvoluted, and 32,829 (89.1%) and 30,749 (89.3%) BACs in the ZS97 and MH63 libraries were tagged, respectively. Using the WGP sequence tags for each BAC clone from each library, two new PMs were constructed with FPC software^13^ (Version 9.4). After manually editing and integration with the previous SNaPshot PMs^11^, the improved ZS97 and MH63 PMs consisted of 539 and 401 contigs, containing 28,372 and 24,519 clones, and had 4,457 and 6,230 clones as singletons, respectively. Total contig sizes were estimated at 342 Mb for ZS97 (N50=940 kb) and 349 Mb for MH63 (N50=1,270 kb).

### PacBio BAC clone sequencing

Minimum tiling path (MTP) BAC clones from each PM were selected automatically with a customized script and re-arrayed manually into MTP library plates designated OSIZBzz (ZS97) and OSIABzz (MH63), and were stored at −80 °C. A total of 4,714 and 4,751 BAC MTP clones were picked for ZS97 and MH63, respectively. The full lists of MTP clones are available in Supplementary Table 1a–b.

For PacBio BAC clone sequencing, MTP BAC clones were inoculated into 96-well deep-well growth blocks, grown overnight at 37 °C, centrifuged to pellet the cells and then stored at −80 °C until use. BAC pools were then created by combining wells from the frozen blocks into one of six configurations: i.e., single row pools (12 BACs per pool), two row pools (24 BACs per pool), four column pools (32 BACs per pool), six column pools (48 BACs per pool), eight column pools (64 BACs per pool), or full plate pools (96 BACs per pool). DNA was then extracted from each pool using a standard alkaline lysis plasmid DNA isolation protocol^14^. In total, 166 (ZS97) and 209 (MH63) pools were sequenced (see our detailed pooling schema in Supplementary Table 2a–b). Using 16 ug of pooled plasmid DNA, PacBio sequencing libraries were produced following manufacturers protocols as described for the 20 kb template preparation with Blue Pippin size selections. SMRT sequencing was performed on a PacBio RSII instrument using P5/C3 sequencing chemistry and 3 h movies.

### Raw read production with PacBio

Subread analysis for both ZS97 and MH63 BAC pool sequences was performed using the PacBio SMRT Portal (Version 2.3.0). For ZS97, data from 227 SMRT cells (which counts redo reactions) was separated and filtered (i.e., using the RS_Subreads protocol, minimum polymerase read length=50 bp, minimum polymerase read quality=75, and minimum subread length=50 bp) resulting in a total of 107.5 Gb of useable sequence data (total number of polymerase reads=11.6 M, polymerase read N50=12.8 kb; total number of subreads=17.7 M reads, mean subread length=5.7 kb, subread N50=8.0 kb). For MH63, data from 317 SMRT cells were treated as above (174 Gb of useable data; 18.2 M polymerase reads, polymerase read N50=12.1 kb; 26.8 M subreads, mean subread length=5.5 kb, subread N50=7.8 kb).

### PacBio data assembly and BAC sequence identification

**Figure 1:**
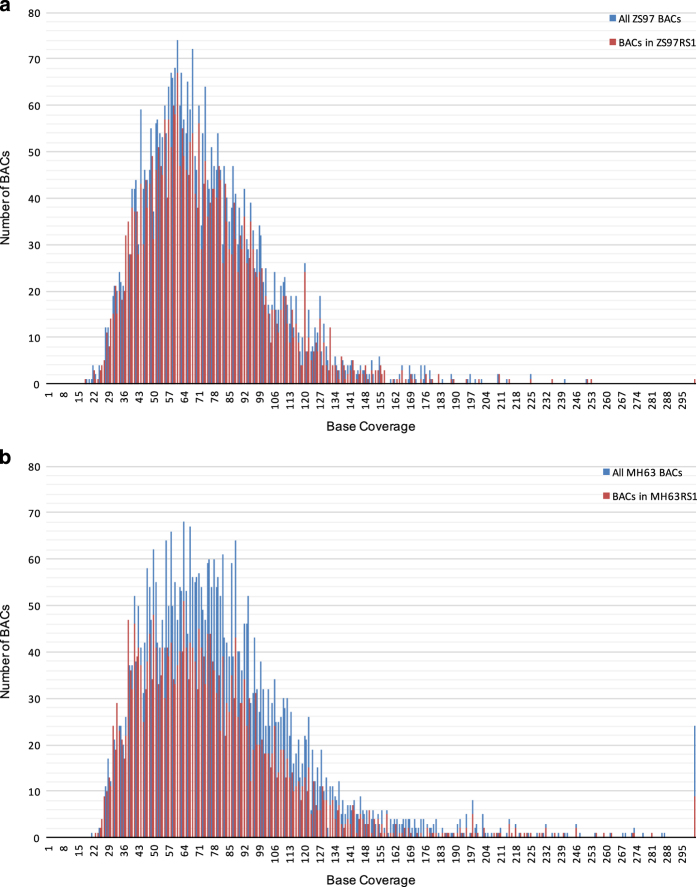
Base coverage distributions of ZS97 (**a**) and MH63 (**b**) BAC sequences.

Sequence data was collected for each BAC pool and assembled independently with PacBio HGAP software (version 2 or 3)^15^ to recover circularized plasmids or BAC specific sequences. In total, 501 HGAP assemblies were run (including assemblies with multiple data sets of the same pool if redos were required) for all sequence pools. Associations between pools and assembly job Ids are shown in Supplementary Table 2a–b. We used a custom pipeline called ‘postHGAP’^16^ to automatically perform circularization and identification of BAC sequences. After running ‘postHGAP’ for each HGAP assembly, we were able to identify 4,571 and 4,488 BAC sequences (4,415 and 4,320 of these were fully circularized) from ZS97 and MH63, respectively. The average lengths of sequenced BACs were 121 kb for ZS97 and 151 kb for MH63. On average, each BAC has about 74–83× PacBio sequence coverage (ranging from 18× to 1747×, Fig. 1a,b), with an overall consensus accuracy of 99.94% (calculation based on all HGAP unitigs, ranging from 99.58 to 99.99%). We also determined the average identity of overlapping regions between two neighboring BAC sequences, which was 99.9787% for ZS97 and 99.9749% for MH63, indicating a high sequence accuracy in this study^10^. Notably, the overall full-circularization rates for BAC pool sequencing were 94% (ZS97) and 91% (MH63). Due to some unavoidable errors or contaminations in MTP clone re-arraying, we identified 125 (ZS97) and 200 (MH63) circularized sequences from non-MTP clones and assigned potential BAC IDs to them with available WGP tag data and/or BES information (Supplementary Table 3a–b).

### Plant material, DNA library construction for illumina sequencing

We also employed Illumina short-read sequencing technology to sequence the ZS97 and MH63 genomes using a whole genome shotgun (WGS) approach. Plant materials were grown in a greenhouse, and 4 week-old seeding leaves were used to extract genomic DNA using standard procedures. Paired-end libraries, including small-insert (~300 bp) and two large-insert libraries (5 kb, 10 kb), were prepared with Illumina’s paired-end and mate-pair kits, respectively (Table 1). At least 5 μg of genomic DNA was fragmented by nebulization with compressed nitrogen gas for the short-insert paired-end libraries. A larger amount of high-quality genomic DNA (10–30 ug) was required for the long-insert mate-pair library construction. Illumina sequencing libraries were prepared following the manufacturer’s protocol. The libraries were sequenced on an Illumina HiSeq 2000 following standard Illumina protocols (Illumina, San Diego, CA). The total amount of raw sequence data generated for each variety was ~97.5 Gb data for ZS97 and ~74.0 Gb data for MH63. After a series of data filtering steps were used to remove artificial reads caused by PCR duplication and adapter contamination, a total ~87.4 Gb of high-quality reads (>200×) for ZS97, and ~67.9 Gb (>170×) for MH63 were obtained (Table 1). Library quality was checked by determining the distribution of insertion sizes and sequence depths. Actual insert lengths were determined by mapping paired-end reads to the *O. sativa* subsp. *japonica* cv. Nipponbare reference genome (henceforth termed the Nipponbare RefSeq)^17^.

### Illumina reads preprocessing and *de novo* assembly

We employed a new hybrid approach by combining *de novo* assembly and reference-based methods^18^ to assemble Illumina reads for each genome. All sequenced reads from ZS97 and MH63 were corrected with Jellyfish^19^ and Quake^20^. Corrected reads were trimmed at their termini if their sequence quality was below 20 using the fastx_tool_kit (http://hannonlab.cshl.edu/fastx_toolkit/index.html), and reads adapters were removed using the cutadpat tool^21^ (https://github.com/marcelm/cutadapt/). The processed reads were then mapped to the Nipponbare RefSeq using BWA^22^. All reads that mapped to a continuous region were collected respectively, and the continuous covered region was defined as a block. The definition of blocks and superblocks are the same as previously described^23^ except that our minimum superblock length was 20 kb, and the overlap between superblocks was 2 kb. We locally *de novo* assembled all the collected reads in a superblock using SOAPdenovo^24^. A series of different k-mers was tested, and the assembled contigs with the largest N50 values were retained. The resulting contigs were assembled by AMOS^25^ using their respective reference chromosomes as a guide. Unmapped reads were re-mapped to the *indica* 9311 genome^26^, and assembled using the same procedure.

Nucmer^27^ was then used to align all supercontigs to the Nipponbare RefSeq. We then checked the coverage and mapping depth of the gaps between the contiguously aligned regions. Mapped reads were selected that bridged gaps in the MH63 and ZS97 genomes which were extended 200 bp on both sides of each gap. All selected reads in these regions were assembled using cap3^28^. The assembled contigs were aligned to the two continuous supercontigs and connected based on their sequence alignments.

To incorporate MH63 and ZS97 specific sequences that were absent in both the Nipponbare and 9311 genomes, we performed whole genome *de novo* assembly using all processed reads with SOAPdenovo^24^, and then aligned the *de novo* assembled scaffolds to the combined supercontigs, and further linked the corresponding supercontigs. Lastly, scaffolding was performed using SSPACE^29^, and gaps were filled with gapCloser (http://sourceforge.net/projects/soapdenovo2/files/GapCloser/).

The final statistics of the WGS Illumina assemblies of ZS97 and MH63 are listed in Tables 2 and 3, and were used to fill gaps between neighboring PacBio contigs.

### Building pseudomolecules in two steps

In the first step, all BAC sequence data was loaded into Genome Puzzle Master^16^ (GPM) to build PacBio-based sequence contigs using physical maps and the Nipponbare RefSeq as reference guides^17^. GPM is a semi-automated pipeline that was developed to integrate logical relationship data (e.g., physical maps) to scaffold sequence contigs into chromosome-scale sequences. As a result, 318 (ZS97) and 216 (MH63) assembled contigs were ordered and oriented, as well as anchored to their appropriated chromosomes, after manual checking, editing and removing redundancies. The final PacBio-based assemblies were composed of sequences from 3862 (ZS97, including 57 non-MTP) and 3254 (MH63, including 77 non-MTP) unique BACs.

Since we used a physical map-based sequencing strategy in this study, the gaps in our WGP physical maps are the main causes of the breaks in the PacBio-based assemblies. Hence, our second step was to fill gaps by integration of Illumina WGS assembly data. However, to minimize the impact of potential misassembles in Illumina data, we only used Illumina contigs that could fully connect two neighboring PacBio contigs in this step. In total, 81 gaps in ZS97 (8,988,328 bp) and 35 in MH63 (3,127,191 bp) were filled with 76 (ZS97) and 35 (MH63) Illumina contig sequences (Supplementary Table 4a–b). As a result, the final hybrid pseudomolecules (ZS97RS1 and MH63RS1) contained 237 (including 2 unanchored) and 181 (including 2 unanchored) contigs for ZS97 and MH63, respectively.

## Data Records

All non-sequence data are available at the iPlant Datastore (Table 4). Both the OSIZBa and OSIABa BAC libraries, or individual BAC clones, can be obtained from AGI’s BAC/EST resource center at www.genome.arizona.edu/orders. BAC end sequences were previously deposited in GenBank under accession numbers KG737749–KG771717 (ZS97, Data Citation 1) and KG702200–KG737748 (MH63, Data Citation 2).

Raw PacBio sequencing data is available at NCBI’s Short Read Archive (SRA) under accession numbers SRP071597 (ZS97, Data Citation 3) and SRP071598 (MH63, Data Citation 4). All Illumina sequencing data can be found under accession number SRP071944 (ZS97 and MH63, Data Citation 5). Due to an unexpected disc error, we lost raw PacBio sequencing data from 57 pools. Fortunately, however, all HGAP runs were archived in the iPlant Datastore under ‘smrt-jobs’ where the filtered subreads of those corresponding pools could be retrieved. Assembled Illumina contig data is available at NCBI assembly under accession numbers GCA_001618795 (ZS97, Data Citation 6) and GCA_001618785 (MH63, Data Citation 7).

The final genome pseudomolecules (Version 1) for each reference genome were deposited in NCBI assembly under accession numbers GCA_001623345 (ZS97RS1, Data Citation 8) and GCA_001623365 (MH63RS1, Data Citation 9).

## Technical Validation

Essentially, each genome equivalent BAC library was freshly grown in copied sets of 384-well plates and three dimensional pooling was performed on the bacterial cells followed by pool growth and plasmid DNA extraction using in-house alkaline lysis chemistry. DNA pools were digested by restriction enzymes (EcoRI/MseI) followed by ligation of pool-dimension oligomers that were designed to specifically locate BAC clone addresses and associate to sequences. After mixed molecule amplification, Illumina sequencing was performed and the resulting data was parsed for 50-bp sequence tag identification to each specific BAC clone address, and for producing bands files as input into FPC. FPC was run under high stringency (HS) settings: first with a ‘tolerance=0 [fixed], Cutoff=1e-15’, then the DQ-option (in 3 steps: Cutoff=1e-18, 1e-21, 1e-24) was employed to split problematic contigs. After the resulting HS PMs were generated, we performed an Ends-to-Ends merge step (Cutoff=1e-9) and the incorporation of remarked singletons to contigs (Cutoff=1e-12) to produce reduced stringency (RS) maps. The WGP RS maps were manually edited by integration with our previous low coverage PMs^11^.

For plasmid DNA extraction, MTP clone plates were thawed and inoculated into deep well blocks containing 1.2 ml of 2XYT+12.5 μgml^−1^ chloramphenicol and grown with shaking for exactly 18 h at 37 °C. The pooled BAC cells were collected, washed with water, and individually prepped to isolate plasmid DNA using standard alkaline lysis with Qiagen P1, P2 and P3 buffers. Following DNA quantification of each pooled sample (i.e., 12–16 ug of DNA/pool) a 20-kb PacBio library was constructed following manufacturer’s protocols, that included BluePippin (Sage Science) size selection of templates, and sequenced in a SMRT cell with P5/C3 chemistry for three hours on a PacBio RS II instrument. Once the sequence was generated, an HGAP (v2 or v3)^15^ assembly run was performed under default settings (minimum polymerase read length=100 bp, minimum polymerase read quality=80, and minimum subread length=500 bp), except ‘Minimum Seed Read Length’ as mean length determined by reads of insert from each SMRT cell and ‘Genome Size’ as estimated lengths of total BACs in a pool. Detailed settings for each run can be extracted from the HGAP job archives at the iPlant Datastore. During ‘postHGAP’ processing, we used fixed parameters for sequence circularization (minOverlap=500 bp, overlapIdentity=95%) and BAC Id assignments (minCloneTagNumber=5, tagMatchIdentity=100%, tagMatchPercent=80%; besMatchIdentity=98% if no WGP tag(s) is available).

In the GPM ‘assemblyRun’ step for building BAC-based sequence contigs, the default parameters for merging two BAC sequences were ‘minOverlapSeqToSeq=1000 bp’ and ‘identitySeqToSeq=99%’, plus the overlaps were required to be at the ends of both sequences. We used the Nipponbare RefSeq^17^ as a reference to assign chromosome numbers to assembled contigs, as well as to order and orientate them. Additionally, only one copy of redundant overlapping sequence was retained in an assembled contig, with no preference on determining which BAC sequence piece was kept. However, non-gapped sequences had higher priority over gapped ones. All contigs were manually checked and edited as needed through the GPM^16^ assembly viewer. When using assembled Illumina contigs to fill gaps between two BAC-based contigs, we only selected the Illumina contigs that could fully connect two neighboring BAC-based contigs, and importantly, such overlaps (‘minOverlapSeqToSeq=1000 bp’ and ‘identitySeqToSeq=99%’) were required to occur at the ends of both contigs. When redundancies were found in these regions, the BAC-based sequence pieces were always retained in the final genome assemblies.

This paper is the first release of the raw data for the assembly of the ZS97 and MH63 *indica* rice genomes, and also provides the first versions of two sets of high quality pseudomolecules to the scientific community. DNA sequencing technologies and sequence assembly programs change rapidly, and the datasets presented here contain multiple types of sequencing reads which can be used to develop new methodologies and software tools as test inputs.

## Additional information

**How to cite this article:** Zhang, J. *et al.* Building two *indica* rice reference genomes with PacBio long-read and Illumina paired-end sequencing data. *Sci. Data* 3:160076 doi: 10.1038/sdata.2016.76 (2016).

## Supplementary Material

Supplementary Tables

## Figures and Tables

**Table 1:** Summary of Illumina read data for ZS97 and MH63.

**Variety & library**	**Raw Reads**		**Clean Reads**		**Filter Percent (%)**	
	**No. of Reads**	**Total length (bp)**	**No. of Reads**	**Total length (bp)**	**No. of Reads**	**Total length**
ZS97_short	338,293,782	34,167,671,982	299,201,824	29,652,703,238	88.44	86.79
ZS97_5 kb	436,436,254	33,169,155,304	345,177,784	25,846,914,440	79.09	77.92
ZS97_10 kb	396,565,650	30,138,989,400	327,334,042	24,554,200,308	82.54	81.47
MH63_short	382,103,532	38,592,456,732	341,947,062	33,482,334,975	89.49	86.76
MH63_5 kb	267,288,070	20,313,893,320	214,879,076	16,135,182,511	80.39	79.43
MH63_10 kb	198,185,740	10,107,472,740	173,925,986	8,866,402,830	87.76	87.72

**Table 2:** ZS97 NGS contig assembly statistics*.

**Chromosome**	**Length(bp)**	**Number**	**N50 (bp)**	**NG50**†	**N90 (bp)**	**NG90**	**Max Contig (bp)**
chr01	41,505,150	1,800	249,739	47	31,557	200	1,560,848
chr02	34,325,857	1,467	266,730	41	37,069	159	884,689
chr03	35,724,790	1,154	287,031	38	50,086	136	1,808,391
chr04	29,991,449	2,303	151,932	53	6,534	451	981,333
chr05	27,896,270	1,635	188,526	42	10,219	243	973,308
chr06	29,165,153	1,755	183,332	47	13,724	210	749,307
chr07	27,477,590	1,958	151,089	48	8,298	277	1,065,432
chr08	26,789,663	1,556	173,772	48	14,332	219	739,850
chr09	21,657,142	1,190	218,997	24	15,452	147	1,087,529
chr10	21,689,031	1,663	129,607	45	6,829	278	871,567
chr11	27,003,295	2,130	126,457	60	5,702	366	527,948
chr12	24,887,049	2,048	141,176	43	5,320	354	628,599
chrUn	1,921,273	1,382	1,904	239	593	1,028	20,713
All	350,033,712	22,041	188,515	507	11,222	2,876	1,808,391

*The statistics are based sequence lengths that are larger than 500 bp.

†The number of sequences with lengths equal to or larger than N50.

**Table 3:** MH63 NGS contig assembly statistics*.

**Chromosome**	**Length (bp)**	**Number**	**N50 (bp)**	**NG50**†	**N90 (bp)**	**NG90**	**Max Contig (bp)**
chr01	41,467,158	2,360	146,312	79	14,985	367	659,130
chr02	34,737,720	1,760	162,664	68	19,298	266	616,724
chr03	35,174,344	1,580	180,406	62	27,156	238	526,233
chr04	32,237,781	2,758	83,872	88	5,715	629	652,894
chr05	27,841,841	1,926	113,801	68	9,194	372	530,915
chr06	28,862,311	2,744	59,335	114	5,123	737	447,621
chr07	27,358,412	2,464	100,207	73	4,994	487	530,282
chr08	25,940,982	1,999	93,823	73	7,300	401	587,588
chr09	21,608,822	1,703	113,836	52	6,627	312	686,925
chr10	21,690,036	2,087	80,081	72	4,308	482	376,283
chr11	27,926,507	2,880	79,019	95	3,592	713	598,709
chr12	25,125,875	2,559	80,004	85	3,845	585	367,314
chrUn	1,784,506	1,316	1,723	257	603	990	20,724
All	351,756,295	28,136	107,523	867	6,550	5,407	686,925

*The statistics are based sequence lengths that are larger than 500 bp.

†The number of sequences with lengths equal to or larger than N50.

**Table 4:** Non-sequence data resources deposited at the iPlant Datastore.

**Data**	Subdirectory*	**Public links**
Physical maps (tag, bands and fpc files)	physical-maps	http://de.iplantcollaborative.org/dl/d/BB2DBCD3-4350-4CD7-BC98-5B4B0A7AE5A6/OSIZBa.ZS97.bands
		http://de.iplantcollaborative.org/dl/d/73625150-224F-4CE9-8C00-4772D396B898/OSIZBa.ZS97.tag
		http://de.iplantcollaborative.org/dl/d/1E744086-A106-403A-B06E-24EF57D8D7B1/OSIZBa.ZS97.fpc
		http://de.iplantcollaborative.org/dl/d/55AFD479-2D65-473D-B662-2DCC1F146DB8/OSIABa.MH63.bands
		http://de.iplantcollaborative.org/dl/d/0FB00443-3DE2-4BB2-A057-7AC107A33569/OSIABa.MH63.tag
		http://de.iplantcollaborative.org/dl/d/7DA7E451-FB7E-4A8A-948E-3E36491E1138/OSIABa.MH63.fpc
MTP clones	mtps	http://de.iplantcollaborative.org/dl/d/15D02C7A-468F-48F1-85CD-D8A6B0599B04/OSIZBzz.ZS97.cloneList.txt
		http://de.iplantcollaborative.org/dl/d/6D786FA7-1EE0-459F-B350-37337CE47563/OSIABzz.MH63.cloneList.txt
Sequencing pools	pools	http://de.iplantcollaborative.org/dl/d/566814A4-28ED-40ED-B266-5CFEE1780168/OSIZBzz.ZS97.libraryPool.html
		http://de.iplantcollaborative.org/dl/d/12325167-C58A-460B-9078-6934AF11658D/OSIABzz.MH63.libraryPool.html
HGAP jobs	smrt-jobs	http://de.iplantcollaborative.org/dl/d/E5C83469-AA51-48FE-A300-CC34BBCB9294/allJobs.txt

*URL: https://de.iplantcollaborative.org/de/?type=data&folder=/iplant/home/shared/agi_data/ZS97MH63.

## References

[d1] GenBank 2013 KG737749–KG771717

[d2] GenBank 2013 KG702200–KG737748

[d3] NCBI Sequence Read Archive 2016 SRP071597

[d4] NCBI Sequence Read Archive 2016 SRP071598

[d5] NCBI Sequence Read Archive 2016 SRP071944

[d6] NCBI Assembly 2016 GCA_001618795

[d7] NCBI Assembly 2016 GCA_001618785

[d8] NCBI Assembly 2016 GCA_001623345

[d9] NCBI Assembly 2016 GCA_001623365

